# Visual working memory and action: Functional links and bi-directional influences

**DOI:** 10.1080/13506285.2020.1759744

**Published:** 2020-05-12

**Authors:** Freek van Ede

**Affiliations:** Oxford Centre for Human Brain Activity, Wellcome Centre for Integrative Neuroimaging, Department of Psychiatry, University of Oxford, Oxford, UK

**Keywords:** Working memory, vision, action, attention, anticipation, planning

## Abstract

Working memory bridges perception to action over extended delays, enabling flexible goal-directed behaviour. To date, studies of visual working memory – concerned with detailed visual representations such as shape and colour – have considered visual memory predominantly in the context of visual task demands, such as visual identification and search. Another key purpose of visual working memory is to directly inform and guide upcoming actions. Taking this as a starting point, I review emerging evidence for the pervasive bi-directional links between visual working memory and (planned) action, and discuss these links from the perspective of their common goal of enabling flexible and precise behaviour.

## Introduction

**Figure 1.**
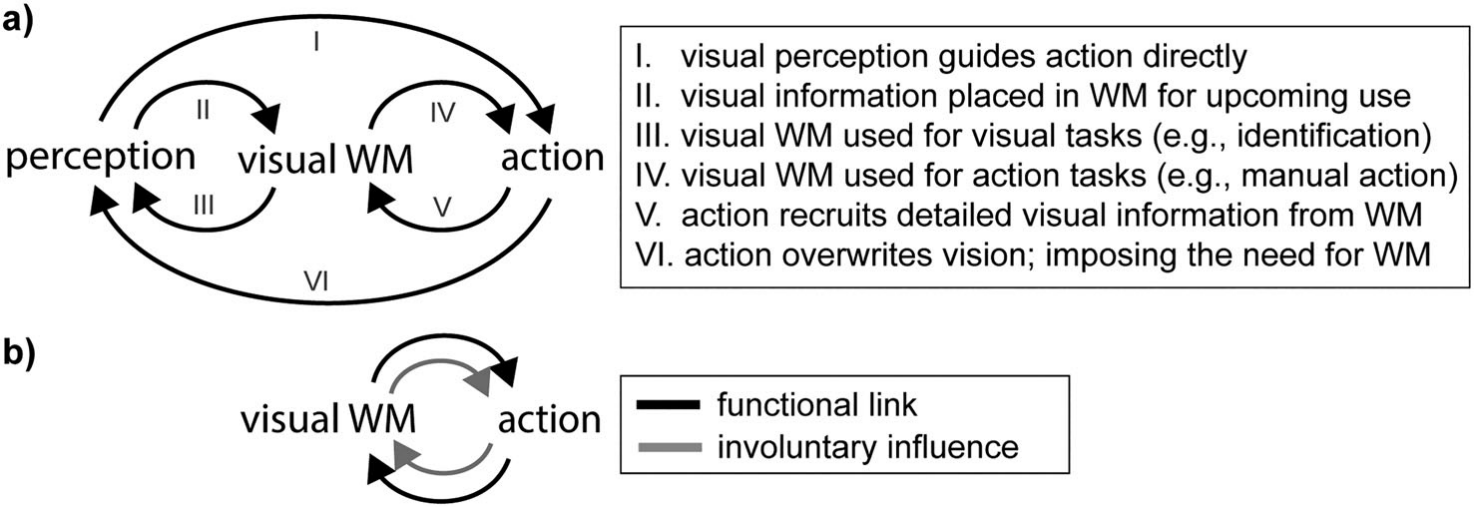
Schematic diagram of routes and influences between vision, visual working memory, and action. (a) Visual working memory here refers to the retention and manipulation of detailed visual information, such as shape and colour. Action refers to overt actions, including eye and hand movements, and encompasses action planning. Working memory is situated at the interface between past vision and future action; when direct route “I” is not feasible because relevant visual information has meanwhile disappeared from sight. Popular laboratory tasks of visual working memory have focused predominantly on route “III”, while research on perception and action has focused mostly on route “I”. This review focuses on routes “IV” and “V”. Route “VI” reminds us that our own actions are often the cause of why we need to rely on visual working memory (when our own movements render visual information “out of sight”). (b) This review is centred on concepts and insights gained from functional and involuntary links between visual working memory and (planned) action. “WM” stands for “working memory”; “perception” refers to “visual perception” within the context of this review.

Visual working memory enables us to hold available those past visual sensations that we anticipate to become relevant for guiding adaptive future behaviour (Baddeley, 1992; Bays & Husain, 2008; de Vries et al., 2020; D’esposito & Postle, 2015; Fiehler et al., 2011; Luck & Vogel, 1997; Miller et al., 1996; Myers et al., 2017; Nobre & Stokes, 2019; Serences, 2016). This situates visual working memory as a key function that interfaces perception and action beyond the immediate (Figure 1(a)), thereby substantially increasing the flexibility of our behavioural repertoire.

With this as a starting point, my primary motivation for writing this short review is that, in the laboratory, visual working memory – here defined as the retention and manipulation of detailed visual information, such as shape and colour – is often studied as a purely visual function (Figure 1(a), route III). While it is evident that visual working memory is *about* vision, it is not only *for* vision. As I will argue, it also serves to directly inform and guide future actions (Figure 1(a), route IV).

Building on a rich literature on direct links between perception and action (e.g., Allport, 1987; Deubel & Schneider, 1996; Gibson, 1979; Goodale, 2011; Hommel et al., 2001) (Figure 1(a), routes I and VI), I here focus on vision-action links that are mediated through working memory. I start from the (related) theoretical perspectives that cognition is fundamentally action oriented (Ballard et al., 1997; Cisek, 2007; Engel et al., 2013; Glenberg et al., 2013; Prinz et al., 2013), and that working memory serves the functional purpose of preparing for prospective behaviour (e.g., Chatham & Badre, 2015; Myers et al., 2017; Nobre & Stokes, 2019; Postle et al., 2006; Rainer et al., 1999; Schneider et al., 2020; Stokes, 2015).

By considering the role of visual working memory for guiding action – and the role of (planned) action in invoking and sculpting visual working memory – we stand to gain greater appreciation of how these two constructs work hand-in-hand towards a common goal of steering flexible adaptive behaviour. This, in turn, may foster relevant cross talk and new experimental approaches at the intersection of both domains, which will be instrumental to breaking new grounds in our understanding of the mechanisms by which working memory enables flexible adaptive behaviour.

To pave the way, I here review recent findings that have begun to converge on the pervasive bi-directional links between visual working memory and (planned) action. These influences can be conveniently sorted into two categories (Figure 1(b)): those focusing on the functional, goal-directed, links between these two constructs; and those reflecting involuntary influences between them. In what follows, I discuss each in turn, followed by a general discussion and outlook.

## Functional links

### Visual working memory serves prospective actions

Visual working memory is conventionally studied in contexts where it serves to guide upcoming task-demands that are primarily “visual” in nature (Figure 1(a), route III) – such as those involving the comparison of a visual probe to the content of memory (delayed match-to-sample or change detection). While such tasks typically require a response (action) at the end of each trial, the responses are dictated by simple task instructions or “action-rules” (Brass et al., 2017; Oberauer, 2013) – e.g., “if match press right button” – rather than being guided directly by detailed sensory memory content. Visual search provides another popular task of visual working memory (Carlisle et al., 2011; Chelazzi et al., 1993; Gunseli et al., 2014). While search too often involves a series of actions (eye movements), in conventional laboratory tasks with multi-item search displays, memory templates for search inform actions only indirectly, by informing *what* to search, not *how* to search. Interestingly, however, when moving to more naturalistic settings, this distinction between what (memory template) and how (search) becomes less applicable, as distinct search templates may promote distinct search strategies. For example, when searching for a clock or a rug, semantic knowledge of the scene (Peelen & Kastner, 2014; Võ et al., 2019) can inform where to search (wall vs floor) or at what resolution to sample (fine-grained vs. course).

In addition to such vision-oriented task demands, I argue that visual working memory plays a similarly important role for guiding actions directly (Figure 1(a), route IV). Everyday examples of such situations include: navigating to your bed after turning of the lights in an unfamiliar hotel room, planning your exit after driving by a road-sign with directions, or directing a shot on goal based on the memorized position and posture of the keeper while focusing on the ball (rendering the “action goal” out of immediate sight).

While action planning has been a prominent aspect of working memory research since its early days (Cisek & Kalaska, 2005; Curtis et al., 2004; Funahashi et al., 1993; Fuster & Alexander, 1971; Ohbayashi et al., 2003; Snyder et al., 1997; Svoboda & Li, 2018), the vast share of this research tradition has relied on delayed response tasks using pure spatial-location memory, void of detailed visual information (such as visual shape information) at that location. Detailed visual information is often relevant for informing and guiding precise actions in everyday life, as in the examples above. While pure location information may be the primary variable for guiding actions with our eyes (informing where to look), bodily actions, such as grasping an object or aiming a shot at a goal, often require guidance from detailed visual shape information at some location. It is here where working memory of detailed visual information is essential – when this information is not available in front of us – but also here where it has received relatively little investigation.

Indeed, in studies of *visual* working memory that tax more detailed visual representation, the possibility of prospective action planning is often deliberately removed from the task (where participants may know the action-rule, but where the appropriate action depends on the unpredictable nature of the probe screen; such as in change detection and continuous reproduction tasks). Such tasks are elegantly titrated for studying the basic mechanisms of “pure retention” in visual working memory. However, it could be argued that such tasks use the probe stage primarily as a “test” of memory and that, by doing so, we are at the risk of failing to appreciate two critical aspects that are relevant to the thinking behind this review. Firstly, what if the mechanisms and strategies of memory retention themselves depend on, and adjust to, prospective task-demands – for which there is good evidence (Boettcher et al., 2020; Gilad et al., 2018; Gunseli et al., 2014; Lee et al., 2013; Lewis-Peacock et al., 2012; Schmidt & Zelinsky, 2017; Serences et al., 2009; van Driel et al., 2017; van Ede et al., 2017; van Loon et al., 2018; Warden & Miller, 2010)? This would imply that retention cannot be understood without also considering future memory purpose. Secondly, given this prospective purpose of working memory, processes of “holding on to the past” may not only themselves be shaped by strategies and future task-demands, but may often also be accompanied by complementary processes of “preparing for the future”. Concurrent preparation of prospective actions alongside visual memory retention provides a clear example, and takes central stage here.

**Figure 2.**
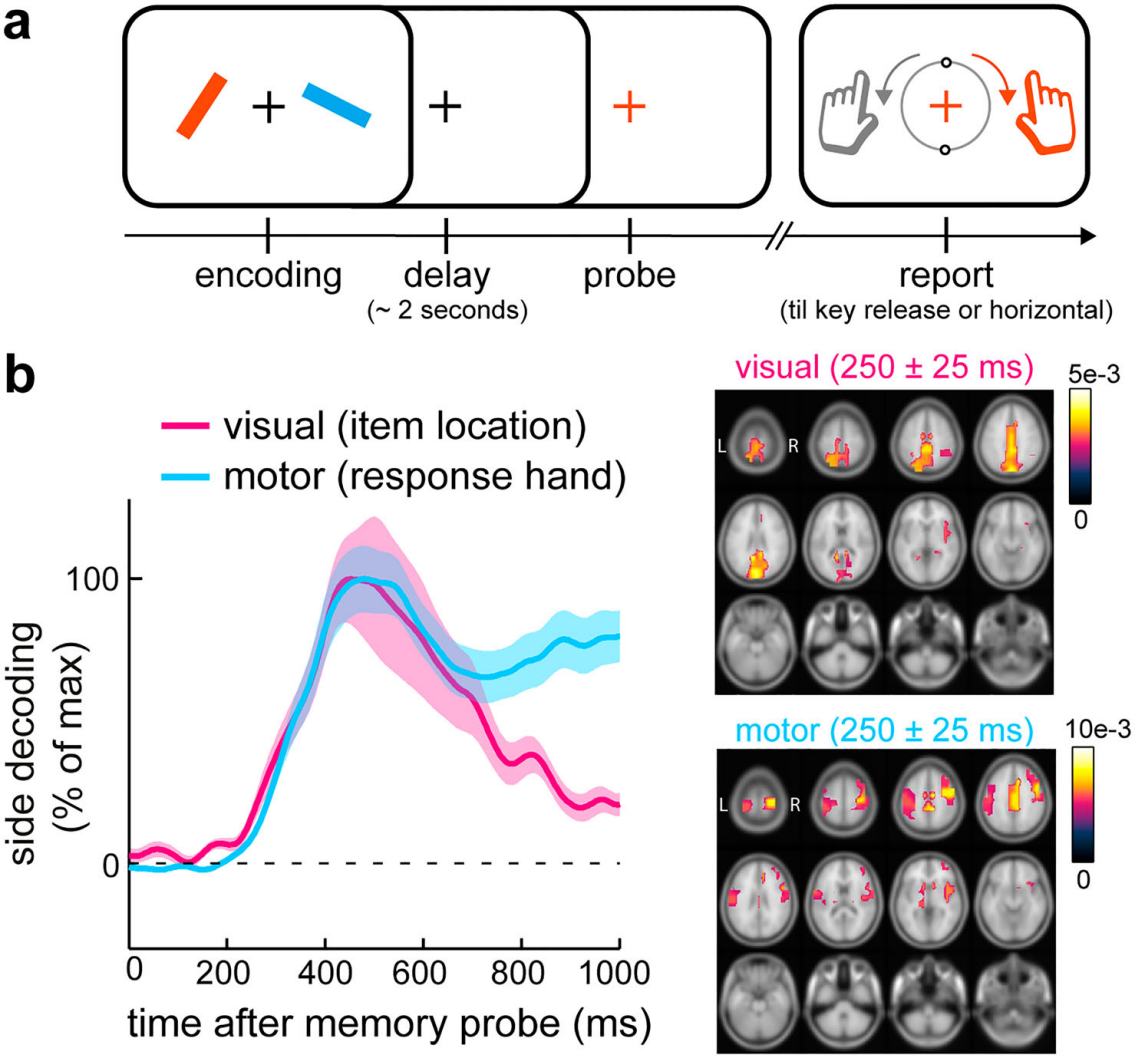
Visual-motor working memory task reveals concurrent accessibility of visual representation and action plans for working-memory guided behaviour. (a) Visual-motor working memory task in which visual shape (orientation) information is linked to specific prospective manual actions (predictable reproduction report) after a memory delay. In this task, actions rely on detailed visual representations from memory. Item locations and prospective response hands (linked to orientation) are independently manipulated to enable independent tracking of visual and motor memory attributes in the EEG. (b) Empirical evidence (EEG decoding) from this task for concurrent selection of visual representations and their associated manual actions from working memory. This data suggest that multiple visual items in memory are held available for selection together with plans for the multiple potential actions they afford. Adapted from (van Ede et al., 2019b).

Several complementary perspective articles (Chatham & Badre, 2015; Myers et al., 2017; Schneider et al., 2020) as well as several recent empirical studies (Boettcher et al., 2020; González-García et al., 2020; Schneider et al., 2017; van Ede et al., 2019b) have started to promote a central role of manual action planning alongside the retention of detailed visual representations in working memory. One clear example of this comes from a recent EEG study of my colleagues and I (van Ede et al., 2019b). In this study, we developed a new visual-motor working memory task (Figure 2(a)) in which we linked visual shape information to specific prospective manual actions (while ensuring the need to hold onto the visual details to guide precise action; and while independently manipulating item location and response hand). Participants memorized two oriented bars whose precise tilt were each associated with a precise manual action (predictable orientation reproduction with the left or right hand). Upon probing either item after a memory delay of approximately 2 seconds, we found that the visual representation (bar location) became selected from memory concurrently with its associated action (response hand), such that visual and motor memory attributes were accessed simultaneously from visual and motor brain areas (Figure 2(b)). This suggests that during the memory delay, participants held two visual representations together with plans for their potential actions. In this way, when either item became relevant (probed), participants could access relevant visual and motor attributes at once, yielding memory-guided action that was not only precise (guided by memorized visual shape information) but also fast. Such prospective action planning alongside the retention of detailed visual information has been confirmed in complementary behavioural (González-García et al., 2020) and EEG (Boettcher et al., 2020; Formica et al., 2020; Schneider et al., 2017) studies.

In our task, the two memory items were always linked to two competing actions (Figure 2(a)). The observation of concurrent selection of visual and motor memory attributes (van Ede et al., 2019b) therefore suggested not only that action planning occurs alongside visual retention, but also that such prospective action planning can take place for more than one memory item at once – linking the notion of *parallel* action planning (Cisek, 2007; Cisek & Kalaska, 2005; Gallivan et al., 2015, 2016) to parallel (i.e., multi-item) visual memory retention. In this light, it is also noteworthy how the number of visual elements can be simultaneously encoded for action has been reported to adhere to similar capacity limits (Gallivan et al., 2011) as those classically reported for visual working memory (Cowan, 2010; Luck & Vogel, 1997). Thus, when we keep multiple visual representations in memory concurrently, parallel action planning can ensure that we are ready for the multiple potential actions that these visual representations may guide.

### Actions invoke visual working memory

As a visual cognition researcher, I have started this review from the perspective of visual working memory and considered the role of prospective action planning herein. From an ecological viewpoint, however, it is perhaps more sensible to start from the perspective of our action goals, and to situate visual working memory herein (Figure 1(a), route V). Our actions and action plans invoke visual working memory in at least two fundamental ways. First, our actions are a key route that impose the need for visual working memory in the first place. As we move around the world, visual information (that may still hold relevance) is rendered invisible as we look away or pass by it (Figure 1(a), route VI). Second, and more directly relevant in the current review, precise actions require detailed visual information. When this information is not currently available to our eyes (for example, because we have looked away; as in the “directing a shot on goal” example above), our actions must rely on the visual information that we had tactfully kept in working memory in anticipation that it will eventually become relevant.

The appreciation that our actions invoke visual working memory has long been made in the context of eye movements. With every eye movement we make, our retinal inputs are overwritten, requiring continuous updating of how our retinal input relates to the external world. Visual working memory has been postulated to play a key role in this process (Aagten-Murphy & Bays, 2019; Irwin, 1991; van der Stigchel & Hollingworth, 2018), though the timescales involved in such trans-saccadic updating (Prime et al., 2011) are much shorter than the multi-second delay periods of typically considered in other popular laboratory tasks of visual working memory.

While the need and purpose of a short-term memory store may be particularly evident for eye-movements, other types of actions may invoke visual working memory too (and this may occur after longer memory delays). Recent fMRI studies have begun to link working memory of visual-shape information to memory-guided manual actions after multi-second delays. Through this work, it has become clear that planning and executing precise manual actions recruit early visual brain areas (Gallivan et al., 2019; Gutteling et al., 2015), even in the absence of current visual stimulation (Fiehler et al., 2011; Monaco et al., 2018; Singhal et al., 2013). This is in line with a call to detailed visual representations from working memory, in which these “sensory” brain areas have been argued to participate (Harrison & Tong, 2009; Pasternak & Greenlee, 2005; Sreenivasan et al., 2014; van Ede, 2018).

These studies on action planning and execution that find involvement of visual processing, nicely complement and converge with the above-described studies on visual working memory that observe concurrent action planning – forming an nice starting point for increased exchange between research on the mechanisms of visual memory, and research on the mechanisms that support planning and control of action.

## Involuntary influences

The functional links between visual working memory and action discussed above are at the heart of this review. Complementary mutual influences that one may describe as “involuntary” or “non-adaptive” – and in some cases “automatic” (but see Carlisle & Woodman, 2011; Foerster & Schneider, 2020; Neumann, 1984; Olivers et al., 2011) – are also informative. The rationale is as follows: if visual working memory and action are fundamentally intertwined in the brain, then these two functions should influence each other, even when such influences are not adaptive to the laboratory task at hand. In other words, it may be difficult or impossible to keep information in visual working memory – and to select and prioritize information from working memory – without affecting (planned) actions, or to act (or plan an action) without affecting what is in visual working memory. Such effects are thus directly informative for the degree to which these two functions are coupled and can give relevant insights into the overlap in the cognitive and neural architectures that support them – in similar vein as the study of such involuntary influences between perception and action (e.g., Baldauf & Deubel, 2010; Corneil & Munoz, 2014; Deubel & Schneider, 1996; Hommel et al., 2001; Novembre et al., 2018; Simon, 1969; van Ede et al., 2015a, 2015b).

Several such influences have become clear over the past years, and the list is likely to grow as we increasingly appreciate the dependencies of these two functions and their shared goals. I highlight the evidence for a selected set of such findings below, sorted according to the direction of influence. I note up front that the vast majority of these findings come from tasks that were primarily “visual” in nature. Moreover, in contrast to the preceding section that focused on *planned* action, the vast majority of research discussed below pertains to *overt* action, as only few studies to date have investigated mutual involuntary links between planned action and visual working memory.

### Visual working memory influences actions

The content of visual working memory can involuntarily affects our actions (Figure 1(a), route IV), with the vast majority of evidence for this to date coming from oculomotor behaviour. For example, task-irrelevant visual inputs whose features (e.g., colour) match the content of visual working memory have been shown to capture attention and to affect eye movements toward them (Bahle et al., 2018; Beck et al., 2012; Foerster & Schneider, 2020; Hollingworth & Luck, 2009; Olivers et al., 2006; Soto et al., 2006) – especially when these representations are in a “prioritised state” ready for upcoming use (de Vries et al., 2020; Olmos-Solis et al., 2017; van Loon et al., 2017). Saccade *trajectories* too have been shown to be affected by the contents of visual working memory. The paths of goal-directed eye movements (in a dual-task setting) tend to curve away from the location of an item that is held in visual working memory concurrently with the secondary saccade task (Belopolsky & Theeuwes, 2011; Boon et al., 2014; Theeuwes et al., 2005). This may serve to “spare” the memory item from interference in brain areas that participate in both visual retention and gaze control.

These studies demonstrate that oculomotor behaviour is dependent on interactions between what is in memory and what visual input occurs (capture findings) or what action output is required (saccade trajectory findings) in the external world. Recent studies demonstrate that oculomotor behaviour can also be triggered *directly* by the process of selectively attending to contents in working memory, in the absence of visual capture probes or secondary action-task demands. These studies have revealed that fixational eye movements (micro-saccades; Corneil & Munoz, 2014; Hafed et al., 2015; Martinez-Conde et al., 2004; Rolfs, 2009) become directionally biased toward the memorized location of the selected memory item (van Ede et al., 2019a), and that pupil size becomes biased by the memorized brightness of a selected memory item (Hustá et al., 2019; Zokaei et al., 2019). Critically, these effects occurred in the absence of any incentives for such behaviour, and may therefore be interpreted as inevitable consequences of the overlap in neural architectures that control our “external focus” (where to direct gaze and how much to dilate our pupil) and that control our “internal focus” (what to select and prioritize in memory).

Curiously, the above studies also uncover that whether the eyes are pulled towards (Johansson & Johansson, 2014; Spivey & Geng, 2001; van Ede et al., 2019a) or pushed away from (Belopolsky & Theeuwes, 2011; Boon et al., 2014; Theeuwes et al., 2005) memorized items in visual memory may critically depend on the exact nature of the task at hand (whether or not involving a dual-task) and/or on the type of eye-movements under consideration (goal-directed saccades vs fixational micro-saccades).

The content of visual working memory may also recruit brain areas that are typically associated with action, without necessarily leading to observable overt actions. For example, retaining images of “manipulable” objects (e.g., hammer) with inherent actions affordance (Cisek, 2007; Gibson, 1979) has been shown to yield stronger recruitment of the “hand area” of the ventral premotor cortex than the retention of images with non-manipulable objects (e.g., house) (Mecklinger et al., 2004). Whether action affordances associated with such visual material also lead to improvements in working memory performance for such visual material remains contested (Downing-Doucet & Guérard, 2014; Pecher, 2013).

### Actions influence visual working memory

In the reverse direction, actions also affect visual working memory (Figure 1(a), route V). Instructions to make eye-movements during a memory delay can impair performance on a concurrent visual-spatial memory tasks (Lawrence et al., 2004; Postle et al., 2006) (see also (Quinn & Ralston, 1986) for related work using arm movements); though eye-movements that may naturally occur as part of memory retention may facilitate such memory (Williams et al., 2013). Such influences may be attributed to the double demands that such dual-tasks pose on the oculomotor system, as this system may participate in both memory retention and eye-movement control (Jonikaitis & Moore, 2019; Merrikhi et al., 2017; Theeuwes et al., 2009; van Ede et al., 2019a).

Complementing these effects on general memory performance, recent studies have addressed whether specific actions affect specific memory items – i.e., whether actions lead us to involuntarily *select* memory items (from among multiple items in memory) at action-congruent locations. In the domain of perception, it has long been known that visual perception is facilitated at locations congruent with (planned) actions (Deubel & Schneider, 1996; Rolfs et al., 2011). Extending this to the domain of working memory, recent studies have shown that goal-directed eye movements made *after* visual encoding can still facilitate performance of spatially-congruent items (here, congruent with memorized item location) (Hanning & Deubel, 2018; Hanning et al., 2016; Ohl & Rolfs, 2017, 2018); see also (Bays & Husain, 2008). This work is also reviewed in more detail in a complementary article by Heuer et al. (2020) in this special issue. This has been shown to occur so automatically that congruent-item benefits persist even when the saccade-congruent item is *less* likely to be probed for report after the memory delay (Ohl & Rolfs, 2017, 2020).

The involuntary influence of action on enhancing item-specific representations in visual working memory extends beyond eye movements. It has been demonstrated that plans for manual pointing movements too can facilitate action-congruent memory items (Hanning & Deubel, 2018; Heuer et al., 2017) – and that this hand-movement-related memory facilitation can co-occur with eye-movement-related memory facilitation at other locations (Hanning & Deubel, 2018) – again extending earlier findings from the perceptual domain (Baldauf & Deubel, 2010).

The above influences thus show that actions can influence memory in an item-specific manner, building on a rich literature on the role of attention on item selection and prioritization in memory (Griffin & Nobre, 2003; Nobre & Stokes, 2019; Souza & Oberauer, 2016). In addition to attentional prioritization at the level of memory items, it has recently become clear that feature-dimensions – that are shared across memory items – can also be prioritized in visual working memory (Hajonides et al., 2019; Niklaus et al., 2017; Park et al., 2017; Pilling & Barrett, 2016; Ye et al., 2016). Such feature-dimension prioritization has been reported to be contingent on actions as well; such that plans for distinct types of manual actions (grasp vs point) facilitate distinct feature-dimensions of visual information in memory (size vs colour) (Heuer & Schubö, 2017); see also (Heuer et al., 2020).

A recent meta-analysis of dual-task interference on visual working memory also converged on an important role for action in shaping visual working memory; revealing that response demands of the secondary task are a critical factor that determine the magnitude of dual-task interference, even when the secondary task is non-visual in nature (Morey, 2018).

Thus, ample evidence exists for bi-directional influences of visual working memory on our actions, and of our actions on visual working memory. I have labelled these findings “involuntary” and “non-adaptive” because they were not adaptive to the laboratory tasks in which they were reported. However, this does not mean these influences will not have adaptive value in everyday life. Instead, such findings remind us how interconnected these two constructs are in their natural contexts, and how they each rely on similar neural computations and brain structures (Jonikaitis & Moore, 2019; Merrikhi et al., 2017; Theeuwes et al., 2005, 2009; van Ede et al., 2019a; Zokaei et al., 2019). Perhaps it is the rudimentary nature of typical laboratory tasks that make such influences appear non-adaptive or disruptive, whereas in more naturalistic settings these effects reflect adaptive consequences of a visual working memory system that is geared for optimal behaviour.

## Discussion

Just like visual working memory is inherently about the past but often serves the future (de Vries et al., 2020; Myers et al., 2017; Nobre & Stokes, 2019; Rainer et al., 1999); visual working memory is inherently about vision, but often serves to guide future action. Here I have reviewed direct links between visual working memory and (planned) action. This has revealed ample, bi-directional, dependencies between these two functions, both in settings where these are clearly functional, as well as where these are not. I argue that the pervasiveness of these influences makes sense if we consider that these two functions – that may appear somewhat remote at first – often share the common goal of guiding flexible adaptive behaviour. This reinforces the notion of visual working memory as a fundamental interface between perception and action that enables us to extend the temporal intervals by which past perception can inform future action and thus to break away from immediate, reflex-driven, behaviour. By holding onto detailed visual memory representations together with their associated prospective actions, the brain ensures that upcoming memory-guided actions are not only flexible; but also fast (action-ready) and precise (guided by visual detail). For a discussion of complementary functional benefits served by mutual interactions between visual working memory and oculomotor action, see also (van der Stigchel & Hollingworth, 2018).

We have seen how visual working memories are held available together with plans for the manual actions they are expected to guide (Boettcher et al., 2020; González-García et al., 2020; Schneider et al., 2017; van Ede et al., 2019b); and how the notion of parallel action planning (Cisek, 2007; Cisek & Kalaska, 2005; Gallivan et al., 2015, 2016) may extend to the situations where we have multiple items in visual working memory (“parallel visual working memory”) (González-García et al., 2020; van Ede et al., 2019b). This work has also made clear that memory retention may often not be restricted to visual representations or motor intentions alone (Colby & Goldberg, 1999; Gilad et al., 2018; Snyder et al., 1997), but instead involve joint visual and motor retention (with the possibility for mutual interaction). This is reinforced by recent studies that have revealed recruitment of visual brain areas for guiding precise manual action, even in the absence of visual input (Fiehler et al., 2011; Monaco et al., 2018; Singhal et al., 2013).

We have also seen how visual working memory and action continue to influence each other, even when such influences are not adaptive (or even disruptive) to the laboratory task at hand (Hollingworth & Luck, 2009; Ohl & Rolfs, 2020; van Ede et al., 2019a; van Loon et al., 2017). Such influences provide perhaps the most striking demonstration of the tight bonds between these two functions, and the overlap in the neural structures and mechanisms that support them. Through such influences, it is as if the brain is trying to tell us that the laboratory tasks in which they occur are too distinct from the type of tasks in which our brains evolved (assuming such influences stem for evolutionary adaptations that are beneficial in naturalistic settings).

As reviewed here, the interface between visual working memory and action has seen a surge of new developments and new insights in recent years. Still, currently only few laboratory tasks exist for studying working memory of detailed visual information in the context of action. Developing new and refining existing tasks for studying the various mutual links between visual working memory, action planning, and action will thus remain an important goal in going forward. In this, it will also be relevant to complement insights from work on eye movements – which have dominated the relevant literature to date, and where the “need” for memory is perhaps more intuitive – with other types of bodily actions, such as manual actions and full-body movements. Actions of the body are harder to measure and require more space, but unlike eye movements (that are predominantly concerned with spatial locations), manual actions are often guided by more detailed visual information such as (memorized) shape.

Research at the interface between visual working memory and action provides an excellent opportunity to increase our understanding of both, and to foster the integration between these two domains that still remain relatively segregated in mainstream psychology and neuroscience. I am hopeful that this review, and the work reviewed in it, will act as a catalyst to their integration and, with this, to our understanding of the mechanisms that support effective flexible behaviour.
